# Supplementary material for: Assessment and Risk Prediction of Chronic Kidney Disease and Kidney Fibrosis Using Non-Invasive Biomarkers
Source: Int J Mol Sci. 2024 Mar 26;25(7):3678. doi: 10.3390/ijms25073678 (PMC11011737; doi:10.3390/ijms25073678)
Supplement: Supplementary file 1 [file ijms-25-03678-s001.zip › ijms-2887341-supplementary.pdf]

Supplementary Table S1. Non-invasive biomarkers for early diagnosis and prediction of CKD progression

Biomarker	Aim	Source	Total number of subjects	Performance	Follow-up time	Reference
KIM-1	Prediction of CKD progression (mGFR decline >10% per year)	urine	229	OR 1.10 (95% CI: 0.71 to 1.83)	21.6 months	34
KIM-1	Prediction of CKD progression (composite of a 50%decline in eGFR or kidney failure)	urine	665	adj. HR 3.0 (95% CI, 1.9 to 4.8)	6.5 years	35
KIM-1	Prediction of CKD progression (ESKD or 40% eGFR decline)	plasma	894	adj. HR 1.17 (95% CI, 1.05 to 1.30)	8.7 years	23
KIM-1	Prediction of incident kidney failure needing replacement therapy (KFRT).	plasma	594	HR 1.52 (95%CI: 1.25,1.84)	6.2 years	24
NGAL	Prediction of renal and mortality end points (40% decline in eGFR, doubling of serumcreatinine, RRT, or death)	serum	139	adj. HR 2.7 (95% CI: 1.3 to 5.8), p=0.012	5.4 (4.7–5.7) years	26
NGAL	Prediction of CKD progression (mGFR decline >10% per year)	urine	229	adj. OR 0.55 (95% CI: 0.33 to 0.89)	21.6 months (IQR, 13.6–24.7)	34
NGAL	Prediction of worsening of kidney function (changes of eGFR during follow-up)	plasma	112	in CKD stage 1 or 2 signifcant diference in eGFR changes according to high and low levels in NGAL	18 months	25
sTNFR1	Prediction of GFR progression and incident CKD (progression of GFR was defined as progression from a higher to lower GFR group, incidence of CKD was defined as having change to a GFR <60 ml/min/1.73 m 2)	serum	1140	progression: OR per SD increase from 1.24 (95% CI 1.01 to 1.53) to OR 1.73 (95% CI 1.44 to 2.08) for both cohorts; incidence: OR per SD increase 1.46 (95% CI 1.14 to 1.86) and OR 2.04 (95% CI 1.61 to 2.43) for both cohorts	5 years	27
CRP	Prediction of CKD progression (all-cause and cardiovascular mortality)	serum	697	all-cause mortality HR 1.56 (95% CI: 1.07 to 2.29); cardiovascular mortality HR 1.94 (95% CI: 1.13 to 3.31)	125 months	28
CRP	Prediction of renal and mortality end points (40% decline in eGFR, doubling of serumcreatinine, RRT, or death)	serum	139	adj. HR 1.4 (95% CI: 1.1 to 1.8)	5.4 (4.7–5.7) years	26
TNF-alpha	Prediction of CKD progression (composite of ≥50% decline in eGFR or onset of ESRD)	plasma	899	adj. HR 1.42 (95% CI: 1.11 to 1.81); P<0.001	6.3 years	30
DKK3	Prediction of short-term eGFR loss (over 12months, >10% decrease of eGFR)	urine	481	IDI 0.013 (95% CI: 0.01 to 0.02), NRI 0.045 (95% CI: 0.01 to 0.08)	over 12 month	39

DKK3	Prediction of CKD progression (50% increase in serum) creatinine, end-stage kidney disease or death)	urine	351	HR 1.91 (95% CI: 1.04 to 3.52) and HR 3.03 (95% CI 1.03 to 8.92) for both cohorts	36 months	40
Biomarker panel of serum creatinine, osteopontin, tryptase, urea, and eGFR,	prediction of CKD progression (composite of a ≥30% decline in eGFR, ESKD)	serum/urine	480	accuracy of 84.3%	minimum of 12 months during follow-up	42
serum biomarkers	Prediction of CKD progression (progression to eGFR <30 mL/min/1.73 m2)	urine/serum	1629	AUC 0.953 for serum biomarkers	5.1 years	43
16 candidate biomarkers	Prediction of CKD progression (mGFR decline >10% per year)	urine	229	AUC 0.722 (95% CI: 0.652 to 0.795)	21.6 months (IQR, 13.6–24.7)	34
serum biomarkers	Prediction of renal and mortality end points (40% decline in eGFR, doubling of serumcreatinine, RRT, or death)	serum	139	AUC 0.81 (95% CI: 0.73 to 0.89)	5.4 (4.7–5.7) years	26
CKD273	Detection of CKD	urine	889	AUC 0.955	NA	45
CKD273	Prediction of Diabetic Nephropathy (progression to eGFR <60 mL/min/1.73 m2)	urine	1014	HR 1.9 (95% CI: 1.3 to 2.7)	6 years	46
CKD273	Detection and prediction of CKD progression (eGFR decline >-5%per year)	urine	522	AUC 0.821	54±28 months	47
CKD273	Prediction of CKD progression (progression from CKD stage ≤2 to ≥ 3)	urine	797	adj. HR 1.30 (95% CI: 0.94 to 1.78)	6.1 years	48
CKD273	Prediction of CKD progression (decline in eGFR of >5 mL/min/1.73 m2/year)	urine	2672	AUC 0.71	3.3 ± 1.1 years	49
CKD273 subclassifier	Prediction of CKD progressio (decline in eGFR of >5 mL/min/1.73 m2/year, by patient with eGFR>70)	urine	1482	AUC 0.81 (95% CI: 0.77-0.85)	3.24	50
CKD273	Prediction of CKD preogression (30% decrease in eGFR from baseline)	urine	1775	adj. HR 5.15 (95% CI: 3.41 to 7.76), p=0.0001	2.51 years	52
Gal-3	Prediction of risk of developing new-onset CKD (defined as eGFR<60 mL/min/1.73 m2 at follow-up accompanied by ≥25% eGFR decline relative to baseline in accordance with recent proposals for quantifying CKD progression; 2)CKD-related hospitalization or death based on ICD-9/10 codes; and 3) ESKD)	plasma	9148	HR 1.75, p<0.001	16 years	32

Gal-3	Prediction of CKD progression (to eGFR <15 ml/min per 1.73 m2 or end-stage renal disease (ESRD))	plasma/serum	841	adj. HR 1.38 (95% CI: 1.01 to 1.80), p=0.044	2.81 and 3.41 years for both cohorts	33
Gal-3	Prediction of CKD progression (defined as 40% decline in the eGFR or ESKD)	urine	280	adj. HR 4.60 (95% CI: 2.85 to 7.71)	3 years	80
HE4	Detection of CKD (eGFR <90 vs eGFR >=90 mL/min/1.73 m2)	serum	249	Increased HE4 concentrations (p<0.0001)	NA	66
SMOC2	Prediction of CKD progression (to ESKD and death)	plasma	434	ESKD per doubling, cohort 1: HR 1.17 (95% CI: 0.92 to 1.48), cohort 2: HR 1.56 (95% CI: 1.02 to 2.39)<br>death, per doubling: cohort 1: HR 1.36 (95% CI: 1.06 to 1.74)	4.4 and 4.1 years for ESKD and 5.7 years for death	72
PEDF	Prediction of CKD progression	plasma	182	per doubling, cohort 1: HR 1.04 (95% CI: 0.77 to 1.40)	4.4 years	72
CDH11	Prediction of CKD progression	plasma	252	per doubing, cohort 2: HR 1.64 (95% CI: 1.02 to 2.63)	4.1 years	72
SMOC2/Cr	Prediction of CKD progression	urine	358	per doubling, cohort 1: 1.11 (95% CI: 1.02 to 1.22); cohort2: 1.26 (95% CI: 1.04 to 1.53)	4.4 and 4.1 years	72
CDH11/Cr	Prediction of CKD progression	urine	358	per doubling, cohort 1: 1.09 (95% CI: 1.00 to 1.20), cohort :2 1.28 (95% CI: 1.05 to 1.57)	4.4 and 4.1 years	72
THBS2/Cr	Prediction of CKD progression	urine	358	per doubling, cohort 1: 1.09 (95% CI: 0.96 to 1.24); cohort 2: 1.21 (95% CI: 0.87 to 1.69)	4.4 and 4.1 years	72
PEDF/Cr	Prediction of CKD progression	urine	236	per doubling, cohort 2: HR 1.22 (95% CI: 1.09 to 1.36)	4.1 years	72

Supplementary Table S2. Non-invasive biomarkers for prediction of drug response

Biomarker	Aim	Source	Total number of subjects	Performance	Follow-up time	Reference
PRE score	Predicttion of long-term cardio-renal efficacy of aliskiren	multi surce	1125	relative risk change of 7.9% (95% CI 2.5 to 13.4) for the cardio-renal endpoint	48 months	53
CKD273	Prediction of response to spironolactone treatment	urine	111	63% (95% confidence interval: 35–79%), as compared with the two other tertiles combined, 16% (17 to 40%) (P=0.011)	16 weeks	54
DKDp189	prediction of response to anti-hypertensive treatment	urine	1032	AUC 0.633 (95% CI 0.561 to 0.701), p= 0.0008) in DIRECT-Protec 2 and AUC 0.60 (95% CI 0.551 to 0.642), p= 0.0074 in the PRIORITY cohort	3 years	55
in silico intervention	the in silico predicted impact of treatment	urine	5585	NA	NA	58

Supplementary Table S3. Non-invasive biomarkers to estimate the degree of fibrosis

Biomarker	Aim	Source	Total number of subjects	Performance	Reference
FPP_29BH	Detection and correlation degree of fibrosis (IFTA >=15% group vs IFTA <10%)	urine	421	AUC 0.840 (95% CI: 0.779 to 0.889); rho = 0.496, p < 0.0001	87
U-C3M/Cr	Detection of fibrosis (Banff score: advanced fibrosis vs. low–moderate fibrosis); Correlation with the percentage level of fibrosis	urine	134	AUC=0.81 (95%CI: 0.72–0.88), p <0.0001; r=-0.58, p<0.0001	78
HE4	Detection of fibrosis in kidney transplant recipients, Correlation with IF/TA grade in kidney transplant recipients	serum	103	AUC=0.857 (95% CI: 0.779 to 0.934); r = 0.7134, p < 0.001	67
LOX	Detection and correlation degree of fibrosis	serum	329	Fibrotic vs non-fibrotic: AUC 0.80 (95% CI: 0.74 to 0.86); moderate-severe vs mild fibrosis: AUC 0.88 (95% CI: 0.82 to 0.95); r = 0.640, p < 0.001	71
DKK3	Detection of fibrosis (severe TA>=25% vs nonsevere TA<=25%; severe IF>=25% vs nonsevere IF<=25%); Correlation with TA grade, and IF grade	urine	108	(In adults) severe TA>=25% vs nonsevere TA<=25%: AUC 0.864 (95% CI, 0.7248–1); severe IF>=25% vs nonsevere IF<=25%: AUC = 0.825 (95% CI, 0.6911–0.9589); fibrosis % rho= 0.71100, p<0.0001, Fibrosis Grade rho= 0.67983, p<0.0001, Tubular Atrophy % rho=0.60571, p=0.0001, Tubular Atrophy Grade rho=0.55802, p=0.0006, IFTA %, rho=0.67884, p<0.0001, IFTA Grade rho=0.63786, p<0.0001	36
miR-21	Detection of fibrosis in kidney transplant recipients (severe IF/TA grade 3 vs IF/TA grades 0, 1 and 2)); Correlation with IF/TA grade in kidney transplant recipients		42	AUC 0.891 (95%CI: 0.792 to 0.989), Multivariate linear regression model with IF/TA grade and estimated GFR: circulating miR-21 levels and IF/TA score (ß = 0.307, p = 0.03), miR-21 levels and a MDRD (ß =0.398, p =0.006)	68
WISP-1	Correlation with renal fibrosis score	serum	77	r=0.475, p=0.0001	69
SMOC2	Association with the degree of kidney fibrosis (categories of IFTA)	plasma	438	per doubling: OR 1.39 (95% CI: 1.10 to 1.76)	72
CDH11	Association with the degree of kidney fibrosis (categories of IFTA)	plasma	438	per doubling: OR 1.20 (95% CI: 1.02 to 1.41)	72
PEDF	Association with the degree of kidney fibrosis (categories of IFTA)	plasma	438	per doubling: OR 1.27 (95% CI: 1.01 to 1.60)	72
CDH11/Cr	Association with the degree of kidney fibrosis (categories of IFTA)	urine	602	per doubling: OR 1.24 (95% CI: 1.12 to 1.38)	72
PEDF/Cr	Association with the degree of kidney fibrosis (categories of IFTA)	urine	602	per doubling: OR 1.13 (95% CI: 1.06 to 1.20)	72
TGFβ	Association with renal morphology (TA, IF, interstitial cellular infiltration, and the sum of scores for mesangial matrix and interstitial fibrosis (assigned as sclerosis index)).	urine	27	at the time of biopsy: interstitial cellular inflammation, r=0.52, p=0.02; 12 months before the biopsy: IF, r=0.86, P=0.01 and the sclerosis index, r=0.84 P=0.02	74
TGFβ	Correlation with tubulo-interstitial fibrosis	urine	39	No correlatrion with degree of tubulo-interstitial fibrosis (r=0.201, p>0.05)	75
PIIINP/Cr	Association with interstitial and glomerular fibrosis	urine	199 (118 kidney biopsy)	association with interstitial fibrosis: based on BANFF criteria, p< 0.005 or based on the color segmentation image analysis software, r= 0.32; p= 0.0007; glomerular fibrosis, r =0.12, p= 0.31	76
PIIINP/Cr	correlation with Interstitial fibrosis in 6-mo protocol biopsy specimens (grade) after transplantation	urine	79	r=0.410, p< 0.001	77
MMP-7/Cr	correlation with fibrosis score (Semiquantitation Scoring of MTS)	urine	102 (30 with kidney biopsy)	r=0.635, p=0.0016	79

Gal-3	Associations with interstitial inflammation, IF, TA	urine	280	per 100 pg/mL: Interstitial inflammation OR 1.229 (95% CI: 1.083 to 1.417), p=0.003; Interstitial fibrosis OR 1.217 (95%CI: 1.076 to 1.394), p= 0.003; Tubular atrophy OR 1.231 (95%CI: 1.086 to 1.416), p= 0.002	80
MCP-1/Cr or MCP-1	correlation with interstitial fibrosis and tubular atrophy.	urine	634	% Cortical fibrosis group: adj. B 0.28, p=0.004 (MCP-1), adj B 0.21, p=0.0005 (MCP-1/ Cr): Number of fibrosis and atrophic tubules foci: adj. B 0.28, p=0.002 (MCP-1), adj B 0.16, p=0.005	81
uromodulin/ Cr	association with interstitial fibrosis/tubular atrophy (percentage of the biopsy affected on the basis of clinical histology reports)	urine	364	IF/TA rho=−0.31 (P<0.001): percentage of total biopsy tissue for IF/TA per two-fold difference in uromodulin (adj.), −2.5% (95% CI: −4.6% to −0.4%) of kidney tissue	83
